# A Synaptic Pruning-Based Spiking Neural Network for Hand-Written Digits Classification

**DOI:** 10.3389/frai.2022.680165

**Published:** 2022-02-24

**Authors:** Faramarz Faghihi, Hany Alashwal, Ahmed A. Moustafa

**Affiliations:** ^1^Machine Listening Lab, University of Bremen, Bremen, Germany; ^2^College of Information Technology, United Arab Emirates University, Al Ain, United Arab Emirates; ^3^School of Psychology, Faculty of Society and Design, Bond University, Gold Coast, QLD, Australia; ^4^Department of Human Anatomy and Physiology, The Faculty of Health Sciences, University of Johannesburg, Johannesburg, South Africa

**Keywords:** deep spiking neural network, back-propagation, synaptic pruning, MNIST database, feature detection

## Abstract

A spiking neural network model inspired by synaptic pruning is developed and trained to extract features of hand-written digits. The network is composed of three spiking neural layers and one output neuron whose firing rate is used for classification. The model detects and collects the geometric features of the images from the Modified National Institute of Standards and Technology database (MNIST). In this work, a novel learning rule is developed to train the network to detect features of different digit classes. For this purpose, randomly initialized synaptic weights between the first and second layers are updated using average firing rates of pre- and postsynaptic neurons. Then, using a neuroscience-inspired mechanism named, “synaptic pruning” and its predefined threshold values, some of the synapses are deleted. Hence, these sparse matrices named, “information channels” are constructed so that they show highly specific patterns for each digit class as connection matrices between the first and second layers. The “information channels” are used in the test phase to assign a digit class to each test image. In addition, the role of feed-back inhibition as well as the connectivity rates of the second and third neural layers are studied. Similar to the abilities of the humans to learn from small training trials, the developed spiking neural network needs a very small dataset for training, compared to the conventional deep learning methods that have shown a very good performance on the MNIST dataset. This work introduces a new class of brain-inspired spiking neural networks to extract the features of complex data images.

## Introduction

**Figure 1:**
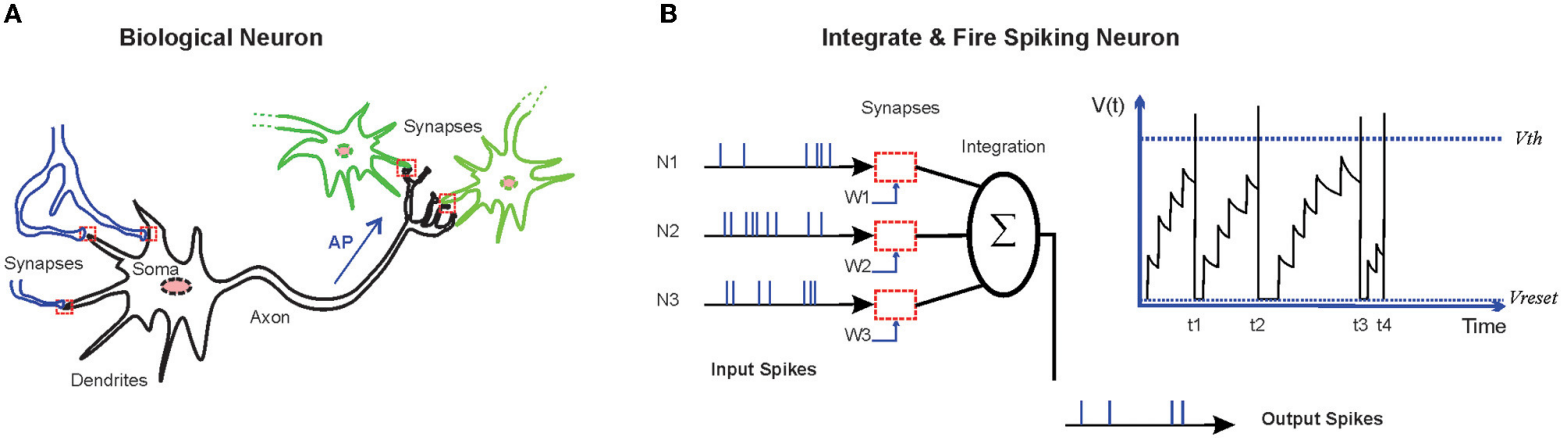
**(A)** A schematic representation of biological neurons. Biological neurons are connected to some other neurons through synapses, where signals are transmitted in the form of released neurotransmitters from the terminal of the axon. The signals from the dendrites are joined in the soma. Action potential (AP), triggered at the soma, travels down the axon and ends at the terminal of the axon. The axon is the elongated fiber that extends from the soma. **(B)** Integrate and Fire neurons are dynamical models of the biological neurons. Incoming spikes push the state of the neuron [membrane potential (V)] higher. The neuron fires at the moment of threshold (*V*_*th*_) crossing. After firing, the state variable is reset to its base value (*V*_*reset*_).

The human brain has demonstrated amazing cognitive capabilities to learn and recognize complex visual patterns in noisy contexts (Kasabov, 2019; Langner et al., 2019). Information processing in the human brain is performed *via* the activation of sensory neurons and subsequently sending the inputs into cortical neurons that lead to complex spiking patterns of neuronal populations to either make a decision or to store the information (Arce-McShane et al., 2018). Cortical neurons are sparsely connected *via* dynamical synapses that can be weakened or strengthened (Waters and Helmchen, 2006; Seeman et al., 2018) by some mechanisms, such as activity-dependent or retrograde signaling from other neurons. Understanding such network architectures and molecular mechanisms, and then implementing them in the artificial systems may lead to brain-like machines that are able to perform complex tasks (Faghihi and Moustafa, 2017; Li et al., 2019; Wu et al., 2022). Biological neurons are composed of dendrites that take up the signals from other neurons, the soma that is involved in information processing, and the axon that passes on the generated action potential (AP) into the terminal synapses of the axon (Figure 1A). These signals seem to carry information to other neurons about the overall state of the corresponding neurons (Brunner and Szabadics, 2016). Activity-induced retrograde messengers, as simple molecules or short peptides, are crucial for the formation of some synapses in some regions of the brain through learning and memory (Suvarna et al., 2016).

During neural development, synapses are over-produced and then eliminated over time. This neuronal event is known as “synaptic pruning.” Developmental synapse formation as an activity-dependent process plays a key role in the function of the healthy brain, where synaptic pruning may be mostly regulated by activity-dependent mechanisms (Chechik et al., 1998; Paolicelli et al., 2011; Südhof, 2018). However, for engineered network design, connections are usually added over time from an initially sparse topology. To design engineered networks, adding connections that will soon be removed is considered wasteful (Navlakha et al., 2018).

The artificial intelligence society has been considering neuroscience as a rich source of inspiration for developing cognitive systems that do complex tasks which the artificial systems are not currently able to perform (Ullman, 2019).

Deep neural networks are artificial neural networks composed of more than two neural layers that are extremely simplified structural and functional analog of cortical networks (Riesenhuber and Poggio, 1999; LeCun et al., 2015). In addition, deep learning models can give explanations and assumptions on how the brain may achieve complicated tasks in uncertain environments (Vogt, 2018). Deep learning models usually perform well on many kinds of data but a large dataset is necessary to train them to produce meaningful results (LeCun et al., 2015).

In the recent years, progresses have been accomplished in neuroscience-inspired algorithms by developing spiking neural networks (SNNs; Hassabis et al., 2017). The SNNs that use different dynamical models of biological neurons are computational models which encode and process information in the time domain. Incoming spikes raise the membrane potential to higher values, and at the moment of crossing the predefined threshold, the neuron generates spike. After firing, the state variable is reset to its base value (Figure 1B). In SNNs, sparse and asynchronous binary signals are used as communication tools and synaptic weights between neurons that are subjected to time and activity dependent parameters. The SNNs have demonstrated capabilities for information processing of data of different sources. In addition, the models based on SNNs have demonstrated the capability to successfully simulate neuronal dynamics underlying the cognitive functions (Deco et al., 2008).

Direct application of back-propagation algorithm in the SNNs as used in convolutional neural networks (CNNs) is a challenge for developing the spiking deep learning methods (Pfeiffer and Pfeil, 2018). One of the attempts of the modern deep learning research is to develop the spiking deep networks by understanding how the networks of biological neurons learn to achieve visual tasks and perform feature extraction (Fu et al., 2012; Tavanaei et al., 2019). One interesting example of human capabilities to recognize noisy and complicated information is the recognition of hand-written digits. The MNIST is a database of hand-written digits, which is currently considered as an evaluation benchmark for deep learning methods and improving the machine learning models (Deng, 2012).

The visual system of a human extracts features from noisy and incomplete information in the images of the dataset. Handwritten digit recognition is critical in some machine learning application, e.g., postal mail sorting and bank check processing. The complexity of the problem arises from some facts; the handwritten digit images are not always of the same size, width, and orientation, so the general problem would be to recognize the similarity between the images of the digit classes (Tavanaei and Maida, 2015).

Different artificial neural network architectures have been applied to the MNIST database for digit recognition, while the best performance has been shown by the deep neural networks (Cireşan et al., 2010). The CNNS have demonstrated the best performance (about 99%) on this dataset (Baldominos et al., 2019; Patil, 2020). Deep SNNs (DSNNs) that are brain-inspired information processing systems have shown their interesting capabilities, such as fast inference and event-driven information processing for image classification (Kulkarni and Rajendran, 2018) including the problem of handwritten digit recognition (López-Vázquez et al., 2019).

Event-driven means that SNNs generate spikes in response to stimulation from other neurons and show very small firing activity when they receive sparse inputs; such a strategy results in power-efficient computing (Thiele et al., 2018). The DSNNs have been developed for supervised, unsupervised, and reinforcement learning paradigms (Frémaux et al., 2013).

The learning rule of SNNs is among the most challenging components for developing deep SNNs because the transfer function of the spiking neurons is usually non-differentiable. This prevents the usage of back-propagation algorithm, which is often used in training artificial neural networks (Lee et al., 2016; Kheradpisheh et al., 2018). To overcome this challenge, some back-propagation algorithms have been proposed by treating the membrane potential of the neuron as a differentiable signal to act analogously with the nonlinear activation functions in the artificial neural networks (Lee et al., 2016) or by using “spike-based learning rule” for rate-coded deep SNNs, where the spike count of each neuron is used as a surrogate for back-propagation (Wu et al., 2019). Other learning rules used in SNNs are based on spike-timing dependent plasticity (STDP; Zhou et al., 2020).

“Few-shot learning” refers to a variety of machine learning algorithms that use a very small amount of training data. Few-shot learning uses machine learning methods to recognize and classify new data after being exposed to few training instances. Several few-shot learning methods that are categorized into “data-bound approaches” and “learning-based approaches” have been proposed (Kadam and Vaidya, 2018). The data-bound approaches use data to find a richer representation of data. These algorithms are based on using prior knowledge to reduce the size of the hypothesis space of the classification task (Wang et al., 2020).

The learning-bound approaches rely on improving the learning processes. Transfer learning (Wu et al., 2020) and the meta-learning approaches (Sun et al., 2019) are the most effective learning approaches for few-shot approaches.

In this work, a SNN-based approach is constructed for the classification of hand-written digits. Specifically, a method to extract features from the images of MNIST is developed and a learning rule is used to train the network as well. The learning rule is combined with a synaptic pruning method resulting in a sparse neural connection between two spiking neural layers. The classification accuracy of the model is evaluated over different model parameters to find the best values of these parameters.

## Methods

The human neocortex demonstrates high performance for pattern recognition tasks by presenting a few training samples while artificial systems often need a very large number of training samples (e.g., deep learning methods). A well-known example of such tasks is the recognition of hand-written digits. In the human brain, the vertical and horizontal patterns, circles, and their combinations that construct digits is learned to assign a digit to a picture (Wyss et al., 2003). The underlying neural mechanisms of learning digits by the brain seem to be fundamentally different from learning algorithms that are used in deep learning methods. In this work, we have developed a DSNN for the MNIST classification that uses simplified synaptic mechanisms and activity-dependent synaptic connections. Unlike prior works, the network is trained with a few training samples. The basic synaptic mechanism used in this work is a proposed learning rule and a simplified model of synaptic pruning.

**Figure 2:**
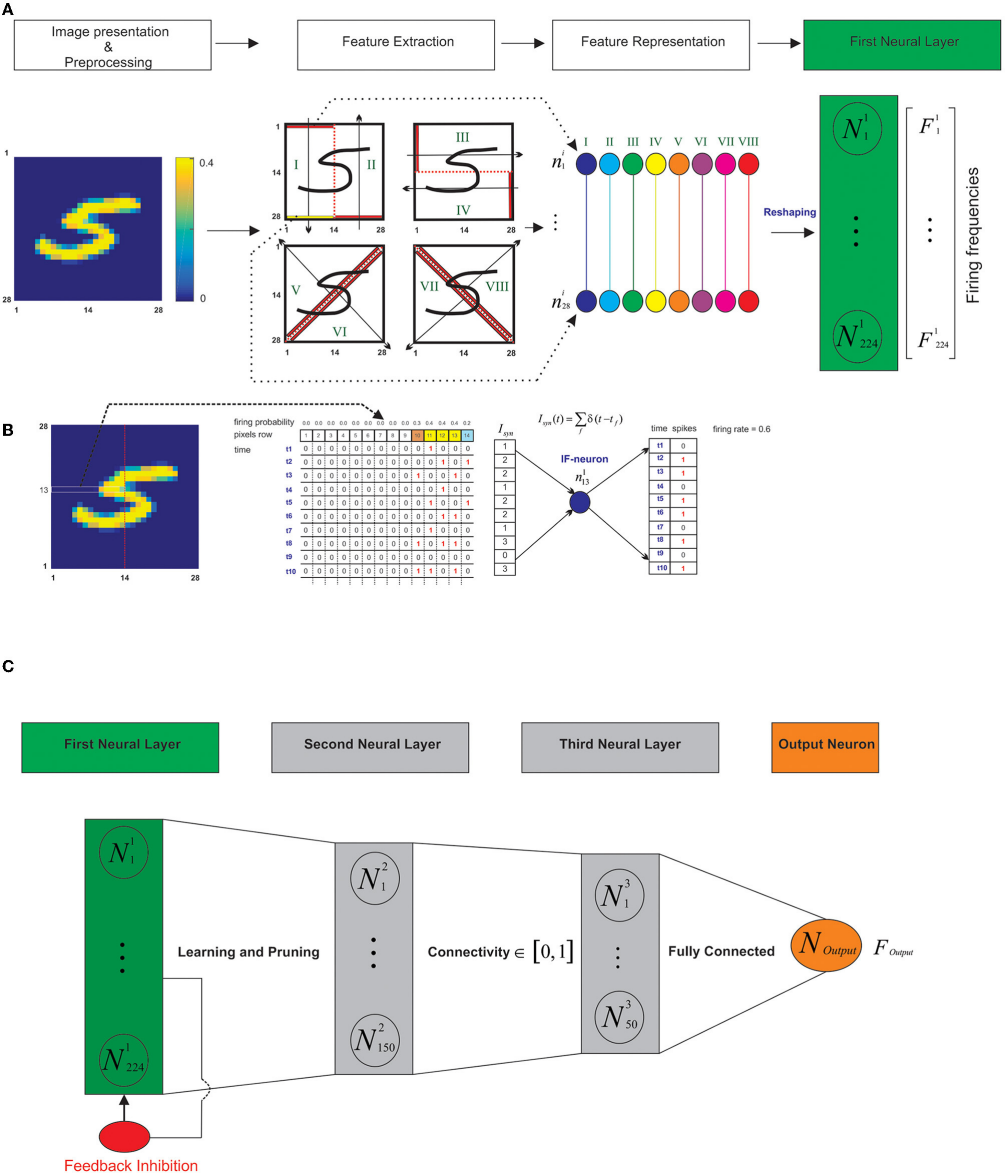
Schematic representation of the architecture of the model. **(A)** The feature representation of the image as spiking neurons in the first layer. After data preprocessing, the image is presented as a matrix of pixels with values between 0 and 0.4. The matrix is divided into eight regions. The information of each region (shown from I to VIII) is represented as 28 spiking neurons. The first spiking neural layer as a feature representation is constructed by concatenation of spiking neurons of the regions as a neural layer with 224 spiking neurons. **(B)** Each pixel of the selected-set of pixels as horizontal, vertical, or diagonal segments are considered as “spike train generators” with mean firing frequency proportional to the intensity of the pixels (here horizontal set of pixels as the 13th row). To represent the information of the pixel as a spiking activity of a probabilistic neuron, a uniform distribution is used to generate vectors composed of sequential 1s and 0s (10 time bins are shown). **(C)** The model architecture. The model is composed of three spiking neural layers and one output neuron (that is fully connected to the third layer). Firing rate of the output neuron is used in the test phase to assign a digit class to the test sample. During the training phase, the proposed learning rule and the synaptic pruning takes place in the connection between the first and the second layers. Different connectivity rates between the second and third layers are used. The feed-back inhibition in the first layer is defined by its parameter value that controls the activity of the network and prevents the over firing of the neurons.

Each written-digit image from the MNIST dataset is a matrix of 28^*^28 pixels (with values between 0 and 255). As data preprocessing the intensity of the pixels is divided by 4 to transform each pixel into a probabilistic neuron that spikes with firing rates between 0 and 63 Hz (0.63 as firing probability corresponding to the maximum pixel intensity; Diehl and Cook, 2015). These probabilistic neurons randomly generate spikes over time (for 1 s in this model) proportional to their firing probabilities (Figure 2B).

In addition, we set the values of the pixels to be equal or higher than 0.4. Different threshold values were used in the experiments; however, no optimal value for the threshold was found. Any threshold between 0.3 and 0.7 compared to other values, resulted in higher classification accuracies. To extract the features of a digit image, each image after preprocessing is partitioned into eight regions by a hypothetical vertical, horizontal or a diagonal line to divide each image equally into halves (Figure 2A, left panel).

Then each pixel of the selected-set of pixels as horizontal, vertical, or diagonal segments are considered as “spike train generators” whose mean firing frequency is proportional to the intensity of the pixels (Figure 2B). To represent the information of the pixel as a spiking activity of a probabilistic neuron, uniform distribution was used to generate vectors that are composed of sequential 1 s and 0 s with length that is set to 1,000 ms (Figure 2B shows 10-time bins of the simulation). Each selected set of pixels in the vertical, horizontal, and diagonal lines are fully connected to a single Integrate and Fire neuron (IF-neuron) where the sum of the spikes is calculated per time bin and is used in the IF-neuron (Equation 1; Figure 2B).

Consequently, the first neural layer is constructed as a neural layer of 224 neurons (28^*^8); about eight regions and 28 neurons in each region are concatenated to construct the first layer (Figure 2A, right panel). The second layer is composed of 150 “IF-neurons.” The number of neurons is arbitrary in this study but we have considered a converging architecture (from 224 neurons to single output neuron). The second layer is fully connected to the first layer with values of initial random synaptic weights between 0 and 1. During the training phase, the weights are dynamically changed according to the learning rule (Figure 2C).

The second layer is connected to the third neural layer that is composed of 50 IF-neurons with a connectivity rate between 0.1 and 1 (as a model parameter). For this purpose, each connectivity value is considered as the mean connection of each neuron in the second layer with the neurons of the third layer. The synaptic weight between the second and the third layers were set to 1. In the connection between the second and the third layers, the synapses are not updated using the learning rule. An output neuron that is also an “IF-neuron” is fully connected to the third layer. The synaptic weight between the neurons of the third layer and the output neuron were set to 1, to provide enough inputs to the output neuron to fire. The firing rates of the output neurons in the trained networks are used in the test phase to assign a digit class to a given image (Figure 2B).

In the training phase, for each digit class, one neural network as described above, is trained. Training is performed by using training samples (with a defined size) for each digit class such that in each experiment, the size of the sets is equal in all the digit classes. In addition, the impact of the size of the training set is studied. For each experiment with a defined training samples size, the experiments are performed 100 times to measure the accuracy of the mean classification.

In the test phase, the entire MNIST test set, composed of 10,000 samples, is used to evaluate the output of the 10 trained networks and the mean firing rate of the output neuron of each trained network is compared to each other to assign the class label to a test sample according to the maximum firing rate of the corresponding output neuron.

In the experiments, the classification accuracy is measured as the ratio between the number of correctly assigned images to a digit class and the total number of images of that digit class in the test data. In this respect, the training set of MNIST provides the required information on the class label of each test image.

Table 1 shows the parameter values used in the simplified IF-neuron model in the neural layers and the output neuron. Equation 1 shows the dynamics of the IF neuron.


(1) $$\begin{array}{r} dV/dt=1/\tau \;\left(\left(E_{L}-V\left(t\right)\right)+I_{syn}\left(t\right)\right) \end{array}$$

$$\begin{array}{r} if\;\;V\left(t\right)>V_{th\;}spike=1\;\&\;V\left(t\right)=V_{reset} \end{array}$$

(2) $$\begin{array}{r} I_{syn}\left(t\right)=\;\underset{f}{\sum }\delta \left(t-t^{\left(f\right)}\right) \end{array}$$

Where δ(*t*) is called the Dirac δ function. It is defined by δ(*t*) = 0 for *t* ≠ 0 and $\overset{\infty }{\underset{-\infty }{\int }}\delta \left(t\right)dt=\;1.$

**Table 1:** The parameters of the IF in the first, second, and third layers and in the output neuron.

τ = 0.01	The membrane time constant
*E*_*L*_ = −0.065	The resting potential (v)
*V*_*reset*_ = −0.065	The reset voltage
*V*_*th*_ = −0.01	The threshold voltage
*V*_0_ = − 0.03	The initial membrane voltage

In Equation 2, *f* indices denote the incoming spikes from the dendrites.

To solve Equation 1 numerically, time-step (*dt*) was set to 0.01 and the initial value of *V* was set to −0.03.

Table 1 shows the neuron parameters as the electrophysiological data of the olfactory system of a honeybee (Sakemi et al., 2021). These parameter values are used for the output neuron except for the threshold value for which different values between −0.03 and 0.03 were used to evaluate the impact of the firing threshold of the output neuron on the maximum classification efficiency of the method.

The spiking activities of each IF-neuron in the first layer are controlled by an inhibitory neuron (Figure 2C); its activity is described by Equation 3.


(3) $$\begin{array}{r} P\left(t\right)=e^{-\frac{\varphi }{∁\left(t\right)}} \end{array}$$

Where *P*(*t*) is the probability to inhibit spike generation by neurons in the first layer at time *t*.

In this respect, a vector with random values of 0 s and 1 s (with the length equal to the number of neurons in the first layer) are generated and used to suppress “1 s” as the neural activity of the neurons of the first layer. The frequency of 1 s is proportional to *P*(*t*).

*The symbol*, φ denotes the “inhibition parameter” that controls the inhibition and ∁ denotes the average activity of neurons of the first layer at time *t*. The role of different φ values induced on the classification efficiency of the model are studied.

To extract image features, an activity-dependent synaptic plasticity rule is developed.

Biological neurons fire in response to dendritic inputs that are received at a given time. A learning rule was developed, in order to select strong presynapses from neurons with high firing rates (compared to the firing rate of postsynaptic neuron) that are involved in stimulating the postsynaptic neuron. This learning rule was developed to extract the important features of a given training image while removing information from the image that is not strong enough to represent the pattern. Figure 2A demonstrates an image of digit class “5” (left panel) and patterns that are desired to be extracted (middle panel). The learning rule allows for the first and the second layers to store the strong information inputs from the image in the synapses.

This learning rule results in sparse connectivity between spiking neural layers that indeed is a method to extract features.

To update the initial random synaptic weights between the first and second layers, the difference of the average firing rate between each pair of neurons in the second layer and the first layer is measured and represented by Δ_(*i, j*)_ (Equation 4). The change in the synaptic weight of the pairs of neurons (Δ*w*_(*i, j*)_) is calculated by Equation 5. This equation allows for a mapping between Δ_(*i, j*)_ and Δ*w*_(*i, j*)_. Although many other equations can be used in this learning rule, the impact of different possible equations on the learning rule and the model is not studied in this work. The parameters of the learning rule determine the rate of weakening or strengthening of the synapses. Therefore, the efficiency of the learning rule strongly depends on a proper range of the value of the used parameters.


(4) $$\begin{array}{r} \Delta_{\left(i,j\right)}=f_{i}^{1}-f_{j}^{2} \end{array}$$

(5) $$\begin{array}{r} {if:\;f}_{i}>f_{j};\;\Delta w_{\left(i,j\right)}=\alpha^{*}\left(\frac{1-e^{-\Delta_{\left(i,j\right)}}}{1+e^{-\Delta_{\left(i,j\right)}}}\right)\;\alpha \in \left[0\;,\;1\right] \end{array}$$

$$\begin{array}{r} {if:\;f}_{i}<f_{j}\;;\;\Delta w_{\left(i,j\right)}=\beta^{*}\left(\frac{1-e^{-\Delta_{\left(i,j\right)}}}{1+e^{-\Delta_{\left(i,j\right)}}}\right)\;\beta \in \left[0\;,1\right] \end{array}$$

*f*_*i*_: firing rate of a neuron in the first layer.
*f*_*j*_: firing rate of a neuron in the second layer.

In the proposed learning rule, α and β are model parameters that are evaluated to find their estimated values for the maximum obtained classification efficiency of the method. In the learning rule, comparing the mean firing rate of a given neuron in the second layer with the mean firing rate of the neurons in the first layer, *either f*_*i*_ > *f*_*j*_ *or f*_*i*_ < *f*_*j*_ plays a critical role in updating the synaptic weights between the given neuron in the second layer with the neurons in the first layer. The training time of the sample presentation (1,000 ms) is divided into four time windows (250, 500, 750, and 1,000 ms), and at the end of each time interval, the synapses are updated using Equation 5.

At the end of presenting the training samples, a threshold (here denoted by μ) is used to set synaptic weights lower than the value of 0 (synaptic pruning). Different threshold values are evaluated to estimate the value of μ to obtain high classification accuracy.

For each digit class, an independent neural network is trained and in the test phase, the test sample is used to evaluate the output of all 10 trained neural networks. Through the test phase, the synaptic weights are not updated and consequently, no synapse is deleted by synaptic pruning. The firing rate of the output neurons of all the trained neural networks are measured and the network with the maximum firing rate is selected to assign a digit class to the test sample.

## Experiments

The developed spiking neural network was used for digit classification using the training set of the MNIST dataset (LeCun et al., 1998).

For this purpose, two experiments were conducted. In one of them, to train the network, training samples were selected randomly from the 60,000 training samples of the MNIST data. To achieve this goal, in each experiment, a different size of the training set per digit class was considered. In another paradigm, the training samples that are similar to the canonical form of the written digits were selected manually. The classification efficiency of the neural network was calculated using 10,000 test samples of the MNIST dataset. Experiments were performed using different parameter values of the learning rule, connectivity rate of the second and third layers, and the threshold of IF neuron model used in the output neuron.

## Results

**Figure 3:**
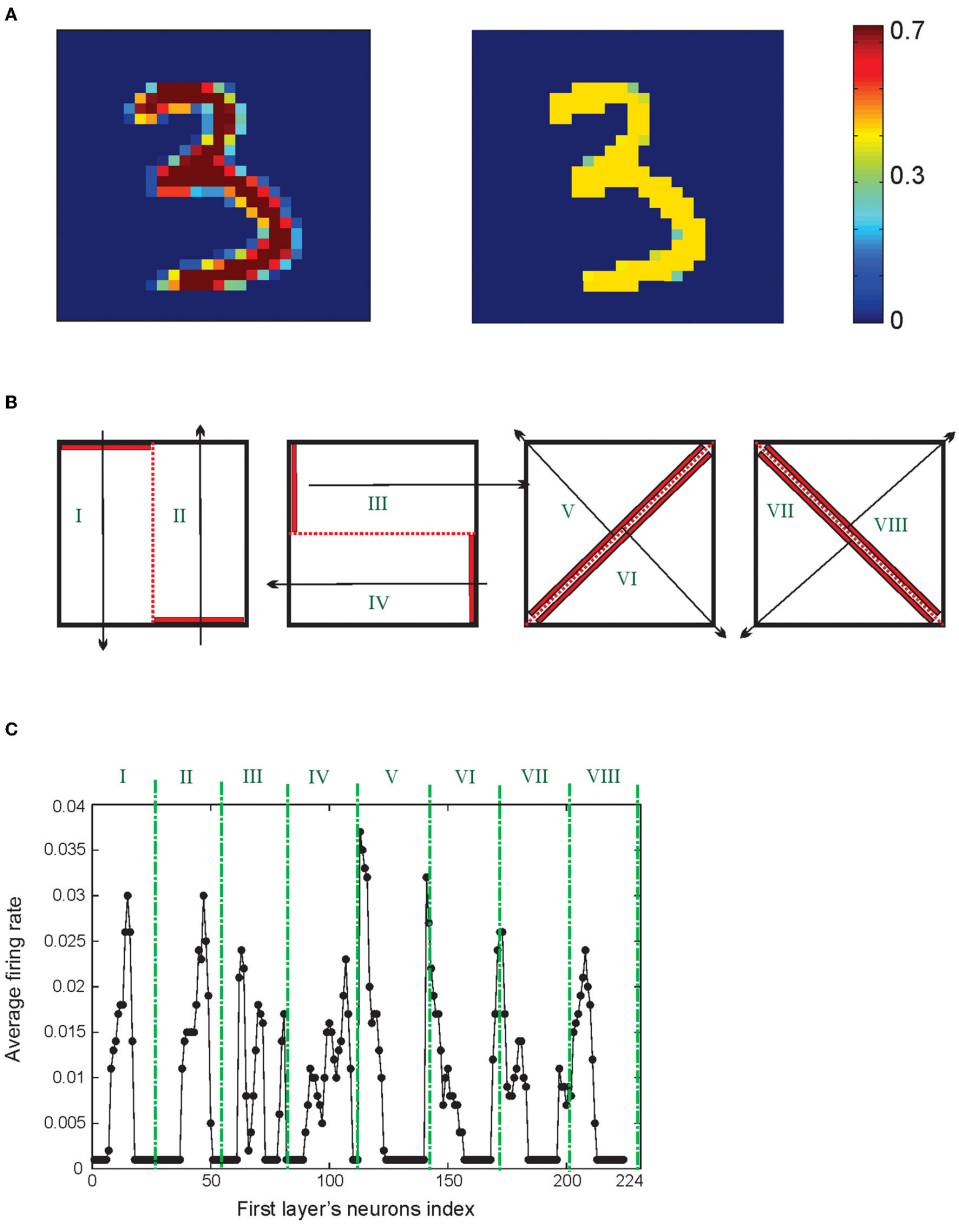
**(A)** Image preprocessing. A hand-written image is used as a matrix of pixel values ranging from 0 to 255 that is normalized between 0 and 0.4. **(B)** Representation of the information of the image as the neuronal activity of neurons in the first layer. For this purpose, matrices of pixels are divided into eight regions. The neuronal activity of 28 neurons of each region is shown in **(C)**.

To use the MNIST dataset to evaluate the classification accuracy of the model, all images are preprocessed such that the pixels get the values between 0 and 0.4 (Figure 3A). Each presented training image to the network stimulates a spiking activity of 224 neurons in the first layer. Average firing rates of these neurons for a sample of digit “3” from the MNIST dataset after partitioning of the data (Figure 3B) are shown in Figure 3C.

**Figure 4:**
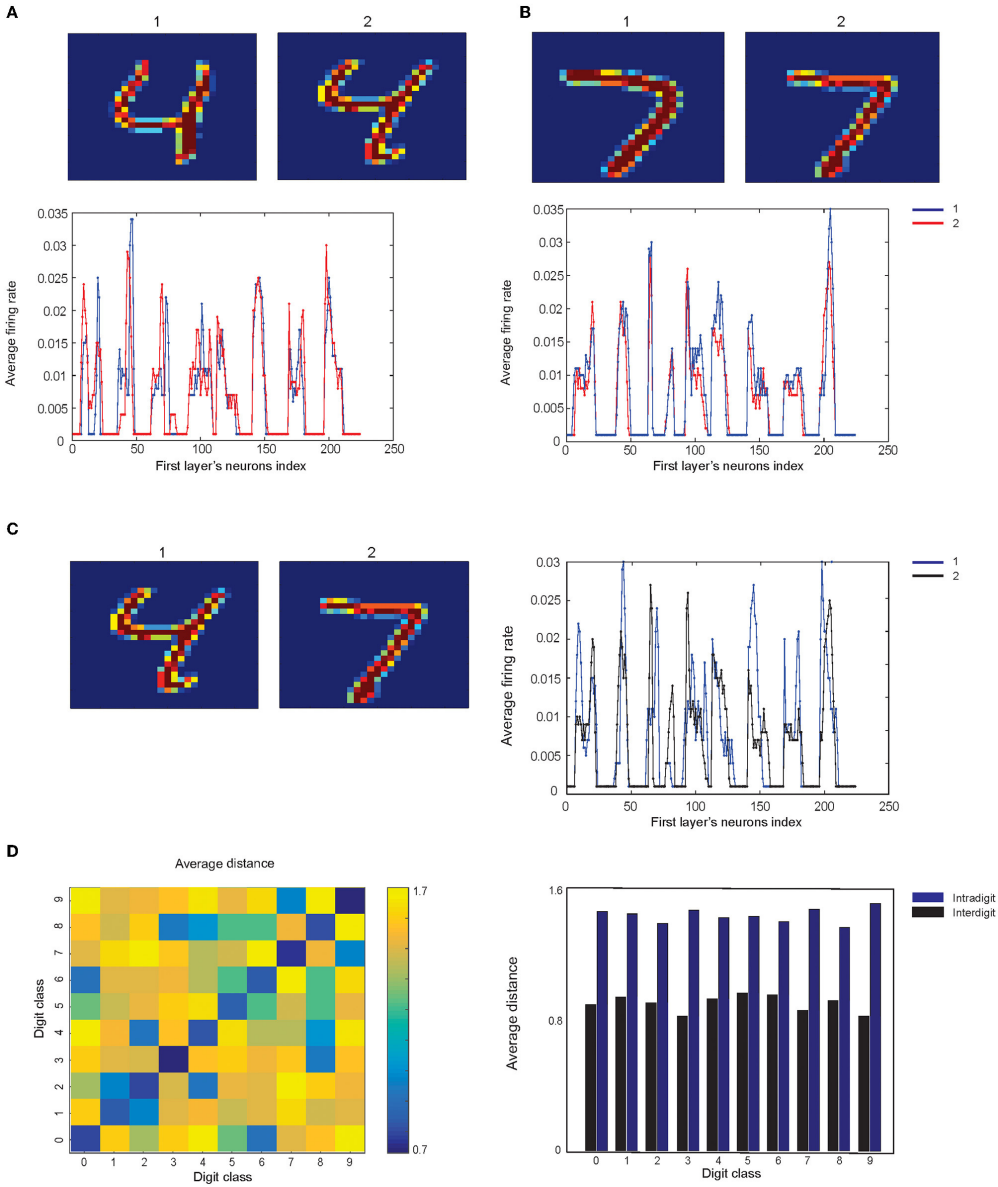
Comparison of the neuronal representation of images from same classes and from different classes. **(A)** Two samples from the dataset are selected for digit “4”'and **(B)** for digit “7”. **(C)** Two samples from digit “4” and “7” and the neuronal activity of the first layer are compared. **(D)** Matrix of the average distance between the pairs of the neuronal representation of training samples. The results show that images from the same digit class have lower distance compared to the samples from different digit classes (right panel).

One can expect that the information of images that are represented as the neuronal activity of the first layer would be as much as possible similar for the samples from the same digit class while showing different activity patterns for different digit classes. In doing so, two images of digit “4” and two images of digit “7” were selected manually from the training set of the MNIST dataset and the pattern of the average spiking rate of the neurons in the first layer for a pair of digits are shown in Figures 4A,B.

The spiking activity pattern of the neurons in the first layer for the images of different classes of digits are shown in Figure 4C. The Euclidian distance of the activity patterns, as a measure of their similarity for pairs of training samples from all digit classes, are shown in Figure 4D.

To achieve this, activity patterns are considered as vectors with the length of 224 and values between 0 and 1. About 100 pairs of samples (for a pair of digit class) are randomly selected from the training data of the MNIST dataset, and the mean of the distances of these 100 pairs of samples are measured and shown in Figure 4D.

The results show that the selected digits from the same class are more similar compared to the images from different digit classes (Figure 4D, right panel).

**Figure 5:**
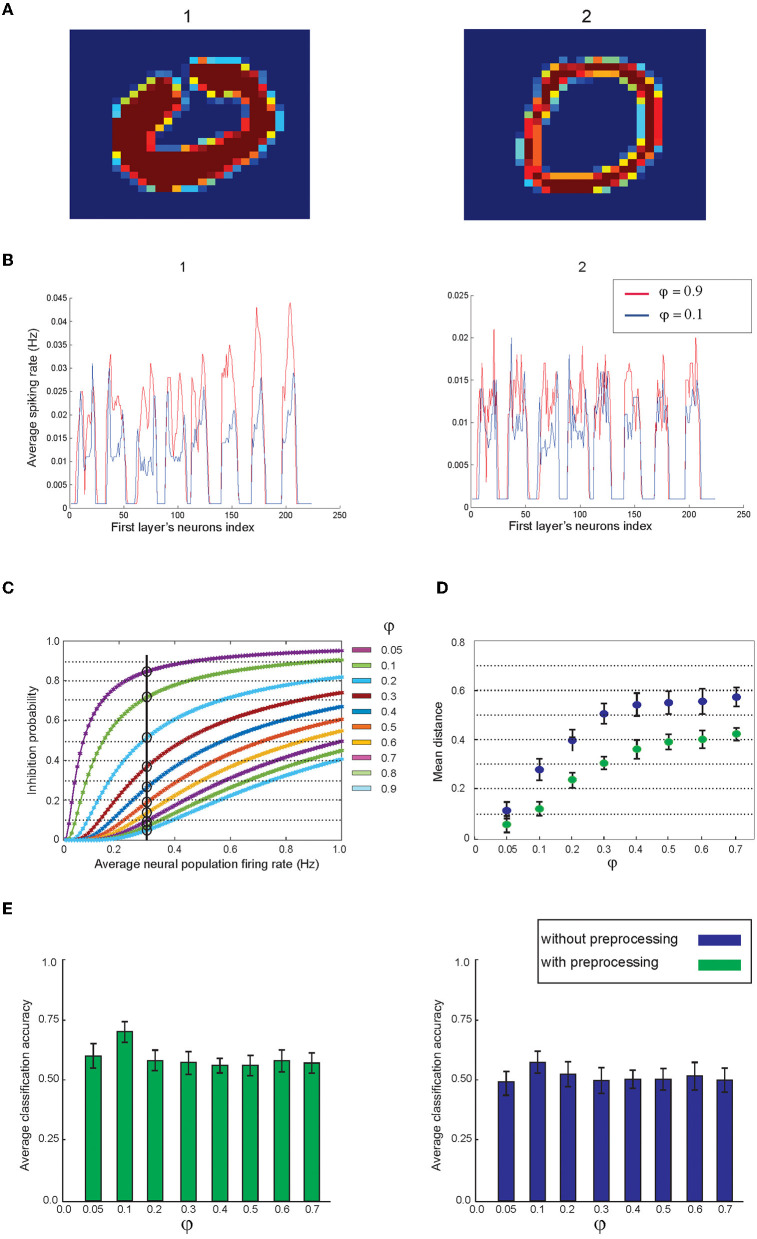
The impact of feed-back inhibition on the neuronal representation of images by the first layer. **(A)** For each digit, there are many samples written thick and this normally makes noise in the pattern of the images from the same class. **(B)** In the model, feed-back inhibition with different parameter values are implemented in the first layer. The figure shows the neuronal representation of two images using low and high inhibition parameter values. **(C)** The dependency of the inhibition probability of the inhibitory neuron on parameter value. **(D)** Lower inhibition parameter values induce higher inhibition on the neural spiking of the first layer that results in a lower distance between the neuronal representations. Two cases are considered: images are used either before or after preprocessing. For each data point, 200 pairs of images per digit class were randomly selected from the training dataset. **(E)** Average classification accuracy using different inhibition parameter values on the entire test data. The right panel shows the results of the mean classification accuracy of all digit classes using 40 “selected training samples” per digit class without preprocessing. The left panel shows the results using 40 “preprocessed” training samples per digit class.

Regarding the variation in the hand-written digits in the dataset where digits are written with different thicknesses and shapes (Figure 5A), the impact of image intensity on the spiking of neurons in the developed spiking network for two written zeroes is shown in Figure 5B. The mean firing rate of the activated neurons in zero images on the right and left panels are 0.23 and 0.13, respectively (φ = 0.9). However, after preprocessing the images, the difference shows a lower value. Feed-back inhibition with different parameter values were used to control the activity level of the neurons in the first layer.

Figure 5B demonstrates the activity pattern of neurons in the first layer for two inhibition values; the lower value leads to more inhibition on the spiking activity of the neurons in the first layer. Figure 5C shows the dependency of inhibition probability on the inhibition parameter (φ) in Equation 3. The lower φ values lead to more inhibition on a given firing rate of the neurons in the first layer (Figure 5C).

The inhibitory activity of feed-back inhibition leads to decrease in the geometric distance of the activity pattern of the first layer where two images from the same digit class are evaluated (Figure 5D). In these experiments, 200 pairs of images per digit class were randomly selected from the training dataset. In addition, the impact of images preprocessed on the distance of neural activities corresponding to these images is shown in Figure 5D. Maximum estimated classification accuracy using 40 selected training samples per digit class was obtained using an inhibition value set to 0.1 and is shown in the left panel of Figure 5E (using preprocessing) and is compared to the results on the training samples without preprocessing (Figure 5E, right panel).

**Figure 6:**
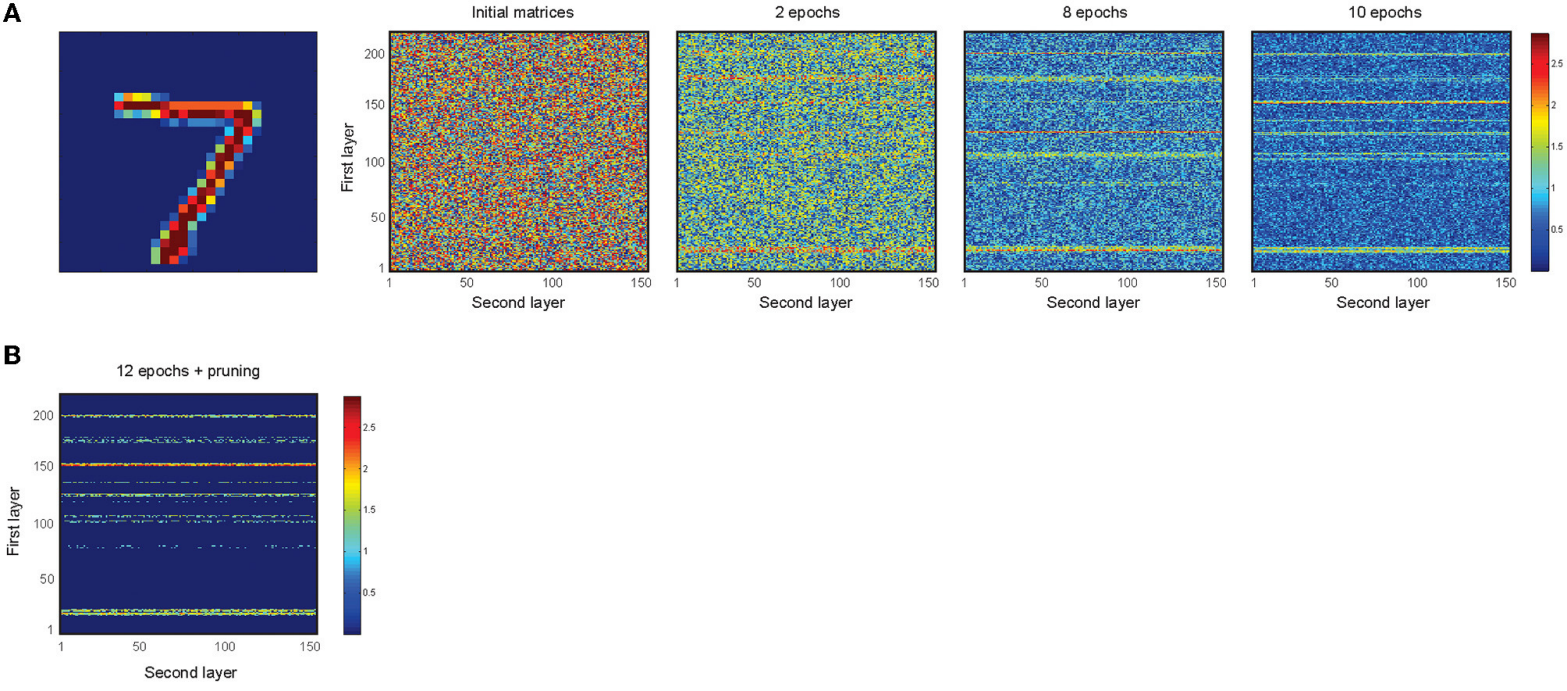
Training of the network for 4 samples of digit class “7” using the learning rule and synaptic pruning. **(A)** The random synaptic weights between the first and second layers are changed according to the learning rule for different epoch numbers of image presentation. Increase in the epoch number leads to gradual changes in the synaptic weights between the activated neurons in the first and second layers. **(B)** A pruning threshold (here 0.8) is used to set weak synapses to 0 such that strong connections of neurons that represent the features of an image are selected.

Figure 6A shows the dynamics of the change in the synaptic weights for an incremental epoch number. To do this, time was considered as 1,200 ms, and divided into 12 equal windows, and 4 training samples for the digit class, “7” were used. At the end of 12 epochs, the connections that have values less than a threshold (here set to 0.8) are set to zero (implemented synaptic pruning) (Figure 6B).

**Figure 7:**
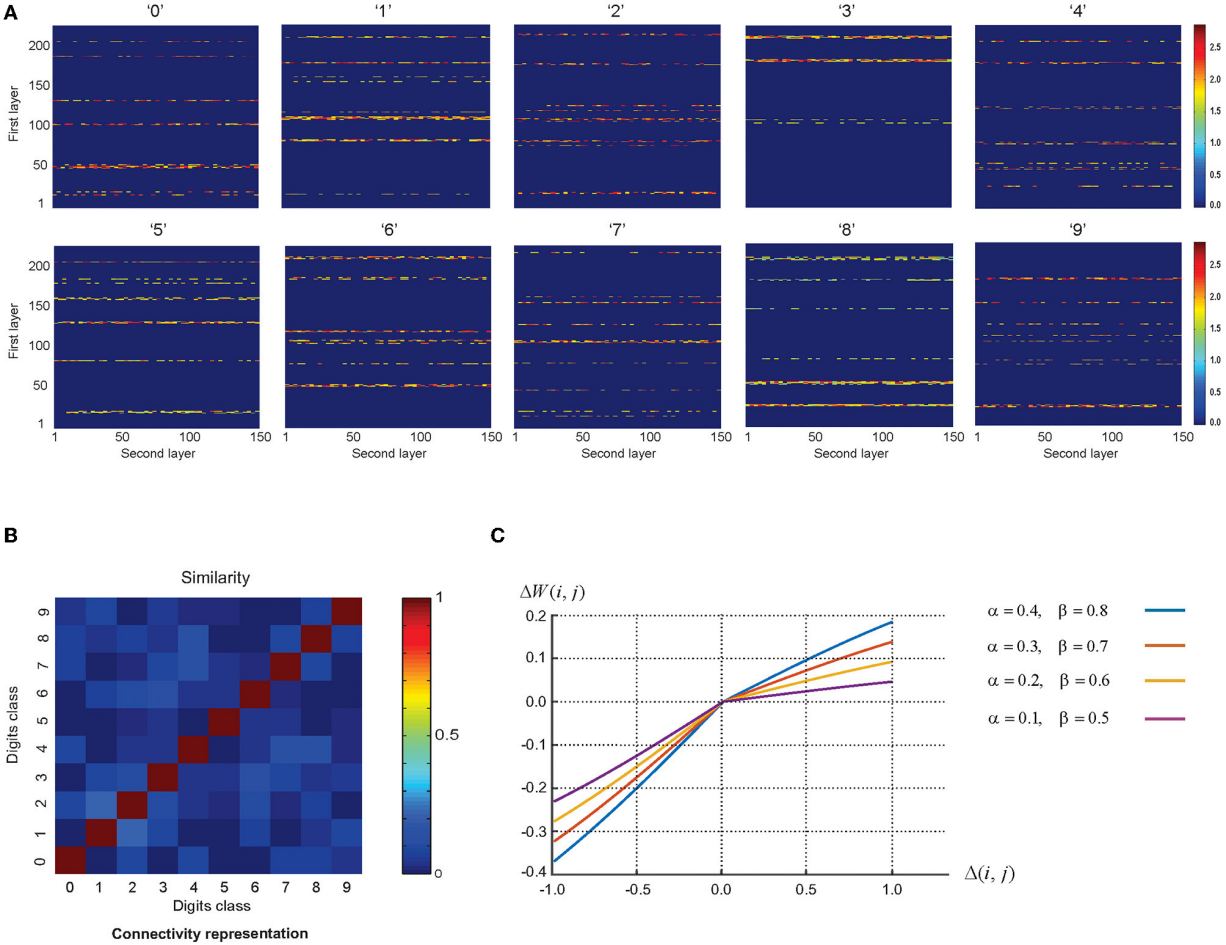
**(A)** Connectivity matrices of the trained networks for digit classes. Connections and their strengths between the first and second layers are shown. One sample per digit class was used and the number of epochs was set to 12. **(B)** The similarity of the connectivity matrices after training by image presentation. The results show remarkable discrimination of the representation of images after training. **(C)** The relation between the change in the synaptic weight of the pairs of neurons (Δ*w*_(*i, j*)_) and the difference of average firing rate between each pair of neurons in the second layer and the first layer (Δ_(*i, j*)_). Among the values of the learning parameters used in the plot, the pair of α = 0.2, and β = 0.6 results in the highest estimated classification accuracy.

For each digit class, the training phase is performed such that eventually 10 connection matrices between the first and second layers are constructed (Figure 7A). These connection matrices named, information channels: constructed in the training phase are used as the extracted features of each digit class. In order to obtain high classification accuracy, the similarity between these connection matrices of the trained networks should be minimized such that each information channel acts specifically to select the corresponding digit class. The similarity between these information channels is updated by using more training samples and the parameter values of the learning rule.

The Euclidean distance of the activity patterns was used as a measure of similarity, which is expressed as Equation 6.


(6) $$\begin{array}{r} S\;\left(A,B\right)=1-d\;\left(A,B\right) \end{array}$$

Where “S” and “d” denote the similarity and the Euclidean distance of pair of patterns (A, B), respectively.

Figure 7B demonstrates the average similarity between the pairs of connection matrices (10 sets per digit class). In the experiments, each image is partitioned into eight regions. Figure 7C demonstrates the dependency of the change in the synaptic weight of the pairs of neurons (Δ*w*_(*i, j*)_) on the difference of the average firing rate between each pair of neurons in the second layer and the first layer (Δ_(*i, j*)_) in Equation 5 using different sets of the values of the learning parameters. Among the values of the learning parameters used in the plot, the pair of α = 0.2, and β = 0.6 results in the highest estimated classification accuracy.

**Figure 8:**
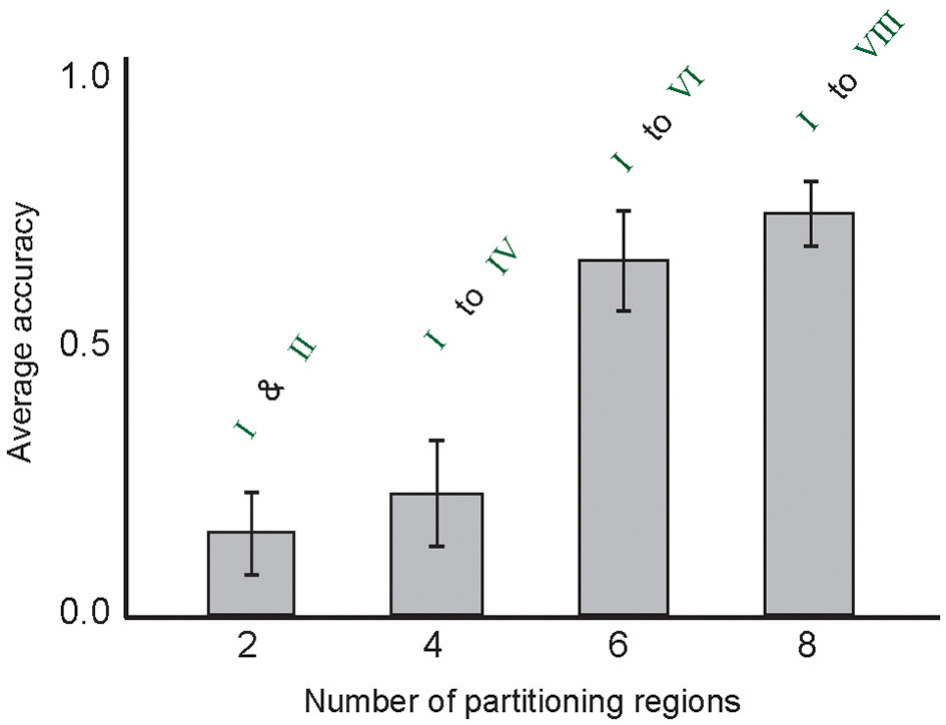
The average classification accuracy of the model on the MNIST test data for the incremental number of used partitioning regions for image representation (from I to VIII). In the experiments, other parameter values were used. The number of selected training samples per digit class was set to 10.

Figure 8 shows the role of the incremental number of partitioning regions on the classification accuracy of the model.

Through the training phase, the synaptic weights between the first and the second layers are changed according to the proposed learning rule and synaptic pruning. Hence, the classification accuracy of the model depends mainly on learning parameters (α, β) and synaptic pruning threshold (μ).

**Figure 9:**
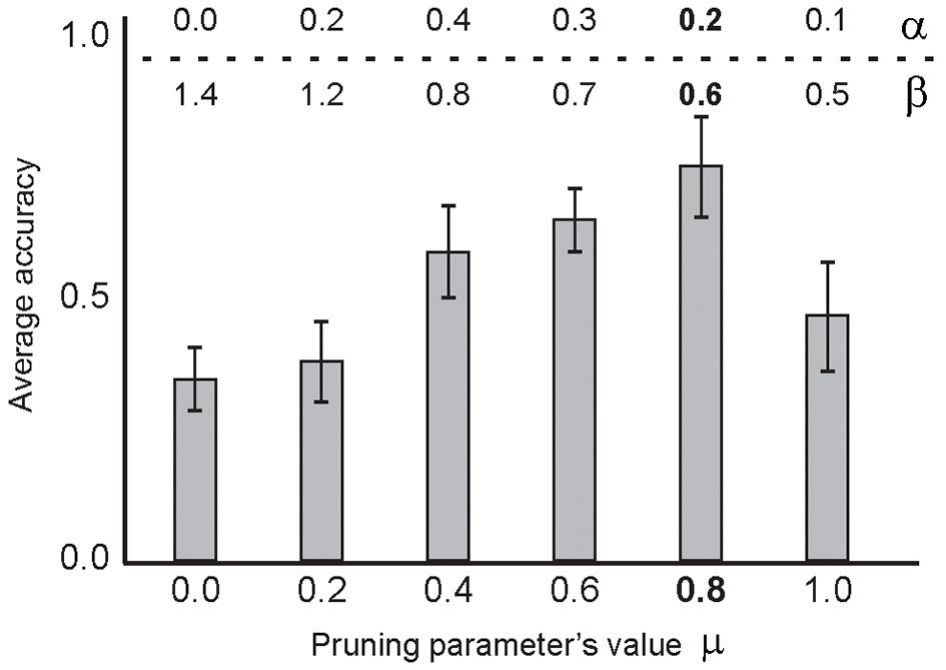
The average classification accuracy for the parameter values of the learning rule (α, β) and the synaptic pruning (μ) parameter values. Average classification accuracy was measured for different pruning thresholds from 0 to 1 after training the network using 10 selected samples per digit class (using 100 experiments per parameter set) and testing on the entire test set. Different learning parameter values were used for a given pruning threshold. The β values from 0 to 2, and α values from 0 to 1 were evaluated. The results show a maximum classification accuracy for the pruning threshold set to 0.8, α = 0.2 and β = 0.6. All possible combinations of α, β, and μ parameter values were used in the experiments and the best results of the parameter sets are shown. In these experiments, 0.01 was used as the firing threshold of the output neuron and 0.2 was used as the connectivity rate between the second and third layers.

**Figure 10:**
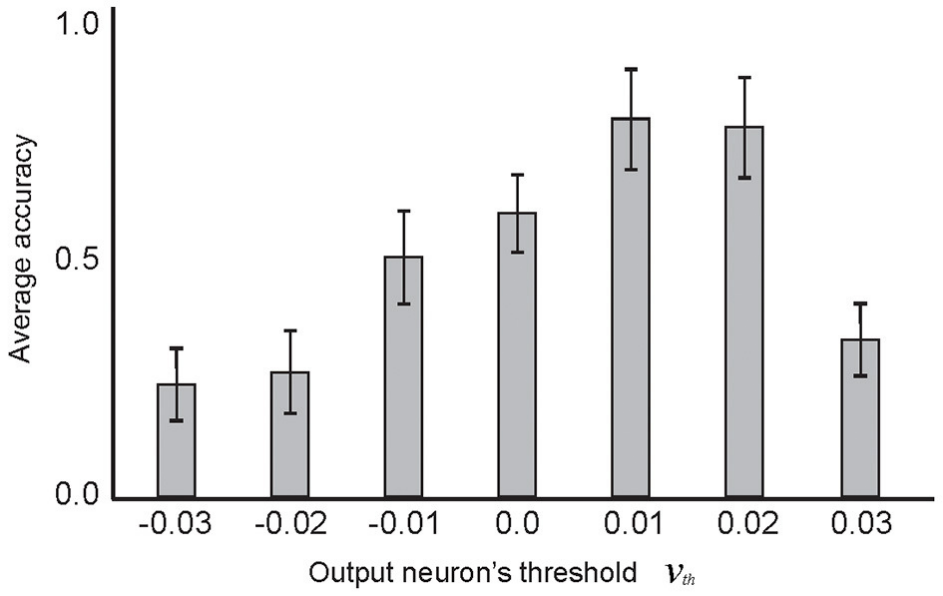
The dependency of classification accuracy on the firing threshold of the output neuron. Average classification accuracy was measured for different firing threshold values from −0.03 to 0.03, by training the network with 10 selected samples per digit class and testing on the entire test dataset. The results show maximum classification accuracy for the firing threshold set to 0.01. In these experiments, α and β values were set as 0.2 and 0.6, respectively. The μ value set to 0.8 and the connectivity rate between the second and third layer was set to 0.2.

Figure 9 shows the selected pruning threshold value (μ = 0.8) corresponding to a pair of learning parameters, α = 0.2 and β = 0.6, for each experiment. The 10 selected training samples per digit class were used and the experiments were performed 100 times. Another parameter of the model is the firing threshold of the output neurons (as used in IF neurons in other layers, Table 1). Figure 10 shows the impact of the firing threshold on the classification accuracy. The selected threshold value, which was 0.01, led to the maximum classification accuracy.

**Figure 11:**
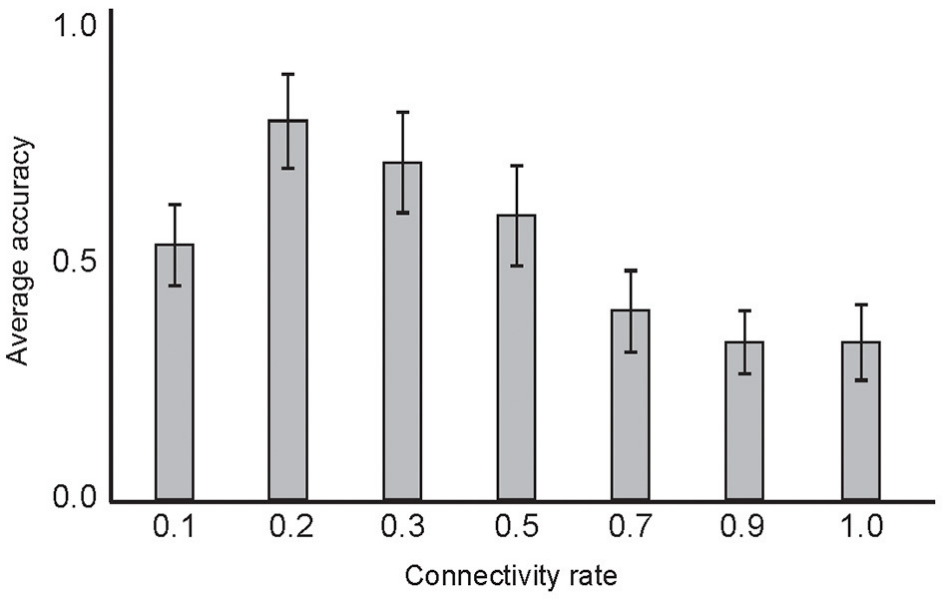
The dependency of classification accuracy on the connectivity rate of the second and third neural layers. Average classification accuracy was measured for different connectivity rates between 0 to 1 by training the network with 10 selected samples and testing on the entire training set. The results show maximum classification accuracy for rates set to 0.2. In these experiments, α, β, and μ values were set as 0.2, 0.6, and 0.8, respectively.

The connectivity rate of the layers in deep network structures may play a role in information processing through network training. Figure 11 shows the classification accuracy of the model using different connectivity rates between the second and third layers. Low connectivity rate equal to 0.2 corresponds to the maximum average classification accuracy in the experiments. In each experiment, 10 selected training samples per digit were used. The experiments were performed 50 times and the mean of the results are shown in Figure 11.

**Figure 12:**
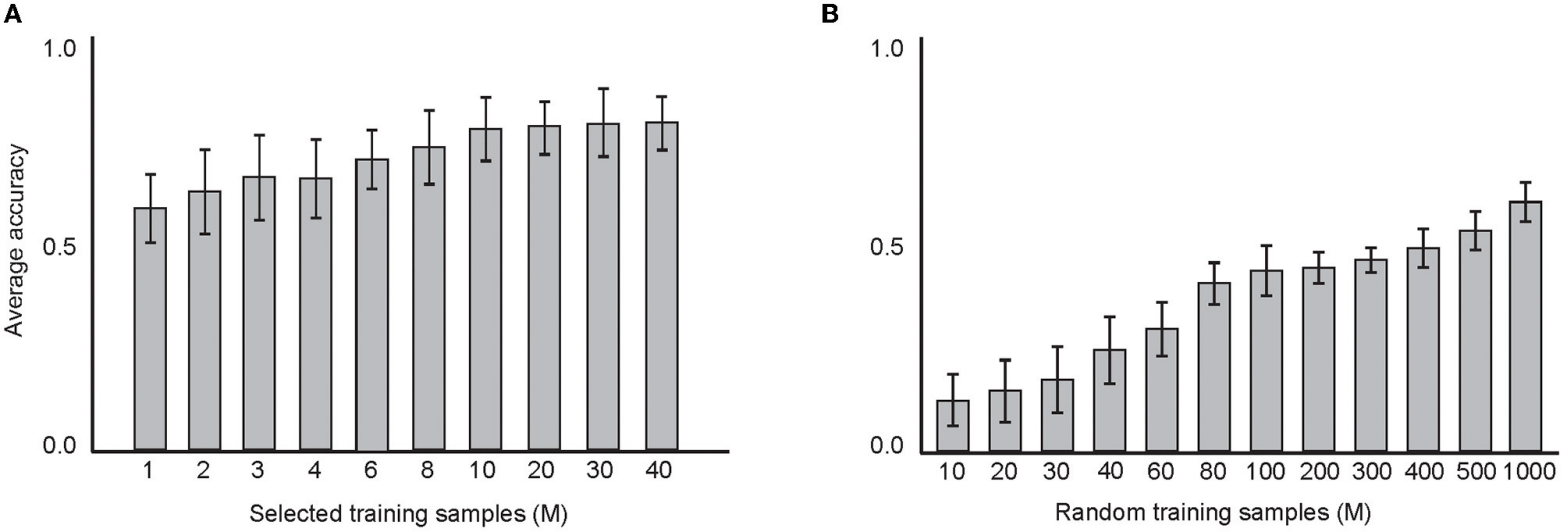
**(A)** The average classification accuracy using “selected training samples per digit class.” About 1 to 40 samples from the training dataset were selected and tested on the test data. A maximum classification accuracy of the model was about 72% using 40 training samples. **(B)** The average classification accuracy using “random training samples per digit class. About10 to 1,000 samples from the training dataset were selected and tested on the test data. Maximum classification accuracy of the model was about 58% using 1,000 training samples.

Figure 12 shows the results on the classification accuracy of the model with estimated parameter values that were measured in the above experiments. Figure 12A shows the results using selected training samples, from 1 to 40 samples per digit and the mean of the classification accuracy over 100 sets of experiments. In Figure 12B, random training samples per digit were used, from 10 to 1,000 training samples per digit. The maximum classification accuracy (about 72%) was obtained when 40 training samples were selected and used in the network training. In these experiments, 10 epochs (each 100 ms) were performed in the network training. The results show the impact of the size of the training set on the classification accuracy.

**Figure 13:**
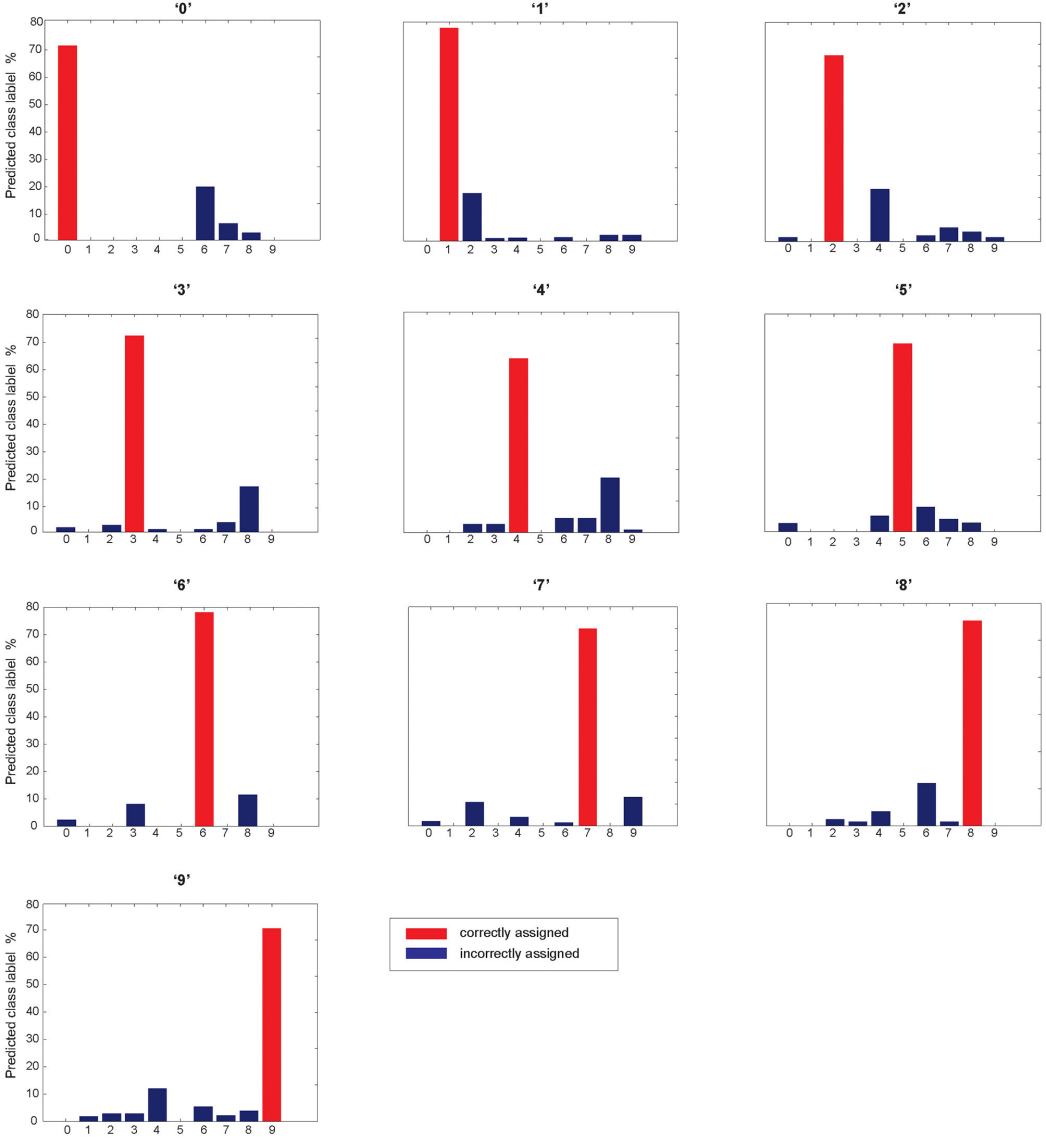
Classification performance for individual digits class. Red bars show the performance of each digit class and blue ones are misrecognized samples assigned by the network. The model demonstrates that digit class “0” is mainly misrecognized with “6”; digit class “1” is misrecognized with “2”; digit class “2” is misrecognized with “4”; digit class ‘3” IS misrecognized with “8”; digit class “4” is misrecognized with “8”; and digit class “8” is misrecognized with “6”.

Figure 13 shows the classification accuracy for each digit class individually tested on the complete test data. The results were obtained using estimated parameter values of the model and 40 selected training samples per digit class that led to 72% accuracy. The classification accuracy above 70% was obtained for digit classes, 0, 1, 3, 6, 7, and 8. In addition, the results allow to observe a distribution of wrongly assigned samples per digit class.

Other SNN models for classification have shown an average accuracy of about 98% on the test samples of the MNIST using the entire 60,000 training samples (Lee et al., 2016; Kheradpisheh et al., 2018) and about 97.2% in another study (Zhou et al., 2020). Higher average classification accuracy on the test data of the MNIST dataset has been obtained in the proposed method (Wu et al., 2019) as 99.26%. Other recent supervised SNNs for the MNIST classification using the entire MNIST training set include methods based on STDP back-propagation with 96.6% accuracy (Tavanaei and Maida, 2019) and temporal back-propagation learning rule (Mostafa, 2017; Zhang et al., 2020; Sakemi et al., 2021) with classification accuracies of 98.1, 98.0, and 97.2%, respectively.

For more details on other supervised SNNs and their classification accuracies on the MNIST dataset and other datasets as well, refer to Kheradpisheh et al. (2020).

One of the few-shot learning methods is supervised non-associative auto-encoder (SNAAE) for the MNIST classification. In this method, stacking layers of auto-encoders (ANNs) are trained in a supervised manner. The performance of SNAAE on the MNIST dataset for 10-shot experiments has been 83.06% while CNNs show 31.52% using 10-shot experiments for each digit class (Gooya et al., 2020).

Recently, some SNNs-based few-shot learning methods have been proposed and tested on some benchmark datasets. In one of these works named surrogate-gradient online error triggered learning (SOEL; Stewart et al., 2020) for online few-shot learning, was tested on IBM Gesture dataset (Amir et al., 2017) or on DAVIS 240C (Lake et al., 2015). This method has shown 64.7% for 1 shot, 65.1% for 5 shots, and 80.2% for 20 shots learning (Stewart et al., 2020).

Our proposed few-shot based classification method as a learning-bound approach selects relevant features of the MNIST images to train the network.

## Discussion

The capabilities of the human brain to learn and retrieve complex and noisy visual patterns while using small training data have inspired researchers and computer scientists to develop artificial systems that demonstrate similar capabilities. These amazing capabilities originate from neuronal architectures and synaptic mechanisms in different neurons in the brain that are partially known and can be used in developing future artificial systems (Faghihi and Moustafa, 2017). Deep learning methods need large amounts of training data in order to show remarkable performance on pattern recognition tasks (Najafabadi et al., 2015). Therefore, developing deep networks that need training data as small as possible is one of the main goals of the modern machine learning researches.

We constructed a novel spiking neural network for hand-written digit classification that extracts image features automatically using a few training samples per digit class. Our work was based on a hypothetical written digit recognition that includes detecting lines and circles that construct written digits. For this purpose, an approach to extract the features of an image was proposed. Each image is represented as neuronal activities of the first layer in the model. In this work, the information of the image is presented as eight regions. The results show the importance of increasing the number of partitioning in improving the classification accuracy.

The results show that there is a correlation between misrecognized digits with a similarity of neuronal representation of digit classes. For example, digit “0” is mainly misrecognized with “6” (Figure 4D), and the distance between their neuronal representations is low, compared to the other distances (Figure 13).

In this work, a firing rate-based learning rule was proposed to train a deep SNN that does not use back-propagation algorithm. After training the networks, a synaptic pruning mechanism was modeled simply by setting weak synapses to zero using some thresholds; in each experiment, only one threshold is used. In this regard, at the end of each epoch of network stimulation, synaptic weights between the first and second layers are updated according to the proposed learning rule. The learning rule compares the firing rates of a neuron in the second layer and a presynaptic neuron in the first layer. If the postsynaptic neuron has a higher rate compared to the presynaptic neuron, the corresponding weight is weakened; otherwise, it is strengthened according to the difference of the mean firing rates of the pre- and postsynaptic neurons. The learning rule allows for detecting and then deleting unimportant synapses which result in the construction of a sparse connection between the spiking neural layers. Using the architecture and mechanisms of the proposed network, we obtained on average, 72% of classification accuracy using 10 samples per digit class for training and corresponding estimated parameter values. However, the efficiency of our method should be evaluated on other types of data for classification problems.

Deep learning methods including CNNs for the MNIST classification started in the 1990's but then for years of research, focused on the support vector machines (LeCun et al., 1998; DeCoste and Schölkopf, 2002). Recently, new CNNs architectures have shown very high classification accuracy on the MNIST dataset that is about 99% but they require the entire training dataset (includes 60,000 samples) and presenting it to the network from 10 to 15 epochs (Wan et al., 2013; Patil, 2020). The SNN-based methods have been developed to extract discriminative features from images for hand-written digit classification. In a study, a two-layer SNN was developed using spike-time dependent plasticity (STDP) along with lateral inhibition and adaptive spiking threshold (Thiele et al., 2018). This method demonstrated 95% of classification accuracy on the MNIST dataset but it required 40,000 training samples. For brain-inspired DSNNs, the importance of sparse connectivity in DSNNs for supervised and reinforcement learning has been recently shown while observing high performance on the MNIST database but using very large training data (Kheradpisheh et al., 2020).

A parameter involved in the classification accuracy of the model is the feed-back inhibition that controls the stimulation of the network in response to images with different intensities. The role of balanced excitation–inhibition in biological neural systems in feed-back and feed-forward inhibitory circuits is known (Yang et al., 2013; Sun et al., 2019). The results have demonstrated the role of strong feed-back inhibition in classification accuracy (Figure 5E). To our knowledge, this is the first study on the role of feed-back inhibition in SNN-based classification methods.

Normal synaptic pruning by microglia cells in the mouse brain is an essential neuronal process and also its contribution to abnormal pruning in the neurodevelopmental disorders have been known (Zhou et al., 2020).

In this work, synaptic pruning helps the network to extract important features of each digit class that are represented as connectivity matrix between the first and second layers. By applying the proposed learning rule and the synaptic pruning model in the training phase of the model, the initially fully connected neurons in the first and second layers have evolved into sparse-connected layers.

The results show maximum classification performance using synaptic pruning parameter value (μ) that is equal to 0.8 when the parameters α* and β* of the learning rule are set to 0.2 and 0.6, respectively. We expect that these parameter values depend strongly on the data type. The image data used in this study is not temporal; therefore, the proposed learning rule should be evaluated for the classification of spatio-temporal data (e.g., electroencephalography data) in which data information strongly depends on the recording time and the source of the location data.

In addition, the electrophysiological values used in the IF-neurons play an important role in the classification accuracy. We have not studied the impact of different sets of electrophysiology parameter values obtained from different biological neurons; only an electrophysiology dataset from a study on the olfactory neurons of an insect were used. The spiking activity of the output neuron was modeled using different values of spiking thresholds.

The results show that using a higher spiking threshold value (set to 0.01) in the output neuron, compared to the value set to −0.01 results in better classification accuracy.

Therefore, studying the electrophysiological parameters of IF-neurons may play an important role in developing bio-inspired SNNs for real applications.

The resulting sparse connectivity matrix constructed after training, named in this work as “information channel,” and its potential for being applied in other deep learning architecture (e.g., convolutional neural networks) need intensive studies. In addition, the connectivity rate between the second and third layers can impact the classification accuracy. The simulations have demonstrated the role of low connectivity rates in the model. To select the training samples from the MNIST dataset, two different paradigms were used, “selected samples” and “random samples.” The results show the impact of selecting training images on the classification accuracy. We think that any better feature extraction method to detect lines and circles that compose the written digits can result in better classification performance.

In conclusion, we suggest that the basic synaptic and network mechanism used in this DSNN performs complex feature selection efficiently with a few training samples and proposes a method to develop a class of machine learning methods. The proposed neuroscience-inspired DSNN has demonstrated good performance to accomplish complicated tasks where the inputs are subjected to noise and uncertainty. We argue that in future, models like the proposed SNN in this study, may play a critical role in developing DSNNs to perform cognitive tasks for modern machine learning techniques.

## Data Availability Statement

The original contributions presented in the study are included in the article/supplementary material, further inquiries can be directed to the corresponding author/s.

## Author Contributions

All authors listed have made a substantial, direct, and intellectual contribution to the work and approved it for publication.

## Funding

This work received financial support from the United Arab Emirates University (Grant No. CIT 31T129).

## Conflict of Interest

The authors declare that the research was conducted in the absence of any commercial or financial relationships that could be construed as a potential conflict of interest.

## Publisher's Note

All claims expressed in this article are solely those of the authors and do not necessarily represent those of their affiliated organizations, or those of the publisher, the editors and the reviewers. Any product that may be evaluated in this article, or claim that may be made by its manufacturer, is not guaranteed or endorsed by the publisher.

## References

[B1] Amir A. Taba B. Berg D. Melano T. McKinstry J. Di Nolfo C.. (2017). A low power, fully event-based gesture recognition system. IEEE Conf. Comput. Vis. Pattern Recog. 2017, 7243–7252. 10.1109/CVPR.2017.78132903824

[B2] Arce-McShane F. I. Sessle B. J. Ross C. F. Hatsopoulos N. G. (2018). Primary sensorimotor cortex exhibits complex dependencies of spike-field coherence on neuronal firing rates, field power, and behavior. J. Neurophysiol. 120, 226–238. 10.1152/jn.00037.2018 29589815 PMC6093958

[B3] Baldominos A. Saez Y. Isasi P. (2019). A survey of handwritten character recognition with mnist and emnist. Appl. Sci. 9, 3169. 10.3390/app9153169

[B4] Brunner J. Szabadics J. (2016). Analogue modulation of back-propagating action potentials enables dendritic hybrid signalling. Nat. Commun. 7, 1–13. 10.1038/ncomms13033 27703164 PMC5059477

[B5] Chechik G. Meilijson I. Ruppin E. (1998). Synaptic pruning in development: a computational account. Neural. Comput. 10, 1759–1777. 10.1162/089976698300017124 9744896

[B6] Cireşan D. C. Meier U. Gambardella L. M. Schmidhuber J. (2010). Deep, big, simple neural nets for handwritten digit recognition. Neural. Comput. 22, 3207–3220. 10.1162/NECO_a_00052 20858131

[B7] Deco G. Jirsa V. K. Robinson P. A. Breakspear M. Friston K. (2008). The dynamic brain: from spiking neurons to neural masses and cortical fields. PLoS Comput. Biol. 4, e1000092. 10.1371/journal.pcbi.1000092 18769680 PMC2519166

[B8] DeCoste D. Schölkopf B. (2002). Training invariant support vector machines. Machine Learn. 46, 161–190. 10.1023/A:1012454411458

[B9] Deng L. (2012). The mnist database of handwritten digit images for machine learning research. IEEE Sign. Proces. Mag. 29, 141–142. 10.1109/MSP.2012.2211477

[B10] Diehl P. U. Cook M. (2015). Unsupervised learning of digit recognition using spike-timing-dependent plasticity. Front. Comput. Neurosci. 9, 99. 10.3389/fncom.2015.00099 26941637 PMC4522567

[B11] Faghihi F. Moustafa A. A. (2017). Combined computational systems biology and computational neuroscience approaches help develop of future “cognitive developmental robotics”. Front. Neurorobot. 11, 63. 10.3389/fnbot.2017.00063 29276486 PMC5727420

[B12] Frémaux N. Sprekeler H. Gerstner W. (2013). Reinforcement learning using a continuous time actor-critic framework with spiking neurons. PLoS Comput. Biol. 9, e1003024. 10.1371/journal.pcbi.1003024 23592970 PMC3623741

[B13] Fu S. Y. Yang G. S. Kuai X. K. (2012). A spiking neural network based cortex-like mechanism and application to facial expression recognition. Comput. Intelligen. Neurosci. 2012, 1–13. 10.1155/2012/946589 23193391 PMC3501821

[B14] Gooya E. S. Al Falou A. Kaddah W. (2020). Robust and discriminating face recognition system based on a neural network and correlation techniques, in International Conference on Image Processing Theory, Tools and Applications (IPTA). (Piscataway, NJ: IEEE), 1–5.

[B15] Hassabis D. Kumaran D. Summerfield C. Botvinick M. (2017). Neuroscience-inspired artificial intelligence. Neuron 95, 245–258. 10.1016/j.neuron.2017.06.011 28728020

[B16] Kadam S. Vaidya V. (2018). Review and analysis of zero, one and few shot learning approaches, International Conference on Intelligent Systems Design and Applications, 100–112. 10.1007/978-3-030-16657-1_10

[B17] Kasabov N. K. (2019). Audio-and Visual Information Processing in the Brain and Its Modelling with Evolving SNN. In Time-Space, Spiking Neural Networks and Brain-Inspired Artificial Intelligence. Berlin; Heidelberg: Springer. 10.1007/978-3-662-57715-8_12

[B18] Kheradpisheh S. R. Ganjtabesh M. Thorpe S. J. Masquelier T. (2018). STDP-based spiking deep convolutional neural networks for object recognition. Neural. Netw. 99, 56–67. 10.1016/j.neunet.2017.12.005 29328958

[B19] Kheradpisheh S. R. Mirsadeghi M. Masquelier T. (2020). BS4NN: binarized spiking neural networks with temporal coding and learning. arXiv[Preprint].arXiv:2007.04039 (2020). 10.1007/s11063-021-10680-x

[B20] Kulkarni S. R. Rajendran B. (2018). Spiking neural networks for handwritten digit recognition—supervised learning and network optimization. Neural. Netw. 103, 118–127. 10.1016/j.neunet.2018.03.019 29674234

[B21] Lake B. M. Salakhutdinov R. Tenenbaum J. B. (2015). Human-level concept learning through probabilistic program induction. Science 350, 1332–1338. 10.1126/science.aab3050 26659050

[B22] Langner R. Eickhoff S. B. Bilalić M. (2019). A network view on brain regions involved in experts' object and pattern recognition: implications for the neural mechanisms of skilled visual perception. Brain Cogn. 131, 74–86. 10.1016/j.bandc.2018.09.007 30290974 PMC6421106

[B23] LeCun Y. Bengio Y. Hinton G. (2015). Deep learning. Nature 521, 436–444. 10.1038/nature14539 26017442

[B24] LeCun Y. Bottou L. Bengio Y. Haffner P. (1998). Gradient-based learning applied to document recognition. Proc. IEEE 86, 2278–2324. 10.1109/5.726791

[B25] Lee J. H. Delbruck T. Pfeiffer M. (2016). Training deep spiking neural networks using backpropagation. Front. Neurosci. 10, 508. 10.3389/fnins.2016.00508 27877107 PMC5099523

[B26] Li J. Li Z. Chen F. Bicchi A. Sun Y. Fukuda T. (2019). Combined sensing, cognition, learning, and control for developing future neuro-robotics systems: a survey. IEEE Trans. Cogn. Dev. Syst. 11, 148–161. 10.1109/TCDS.2019.2897618

[B27] López-Vázquez G. Ornelas-Rodriguez M. Espinal A. Soria-Alcaraz J. A. Rojas-Domínguez A. Puga-Soberanes H. J.. (2019). Evolutionary spiking neural networks for solving supervised classification problems. Comput. Intell. Neurosci. 2019, 4182639. 10.1155/2019/4182639 31049050 PMC6458934

[B28] Mostafa H. (2017). Supervised learning based on temporal coding in spiking neural networks. IEEE Trans. Neural Netw. Learn. Syst. 29, 3227–3235. 10.1109/TNNLS.2017.2726060 28783639

[B29] Najafabadi M. M. Villanustre F. Khoshgoftaar T. M. Seliya N. Wald R. Muharemagic E. (2015). Deep learning applications and challenges in big data analytics. J. Big Data 2, 1–21. 10.1186/s40537-014-0007-7

[B30] Navlakha S. Bar-Joseph Z. Barth A. L. (2018). Network design and the brain. Trends Cogn. Sci. 22, 64–78. 10.1016/j.tics.2017.09.012 29054336

[B31] Paolicelli R. C. Bolasco G. Pagani F. Maggi L. Scianni M. Panzanelli P.. (2011). Synaptic pruning by microglia is necessary for normal brain development. Science 333, 1456–1458. 10.1126/science.1202529 21778362

[B32] Patil P. (2020). Handwritten digit recognition using various machine learning algorithms and models. Int. J. Innov. Res. Comput. Sci. Technol. 16, 337–340. 10.21276/ijircst.2020.8.4.1633229549

[B33] Pfeiffer M. Pfeil T. (2018). Deep learning with spiking neurons: opportunities and challenges. Front. Neurosci. 12:774. 10.3389/fnins.2018.00774 30410432 PMC6209684

[B34] Riesenhuber M. Poggio T. (1999). Hierarchical models of object recognition in cortex. Nat. Neurosci. 2, 1019–1025. 10.1038/14819 10526343

[B35] Sakemi Y. Morino K. Morie T. Aihara K. (2021). A supervised learning algorithm for multilayer spiking neural networks based on temporal coding toward energy-efficient vlsi processor design, in IEEE Transactions on Neural Networks, and Learning Systems. Piscataway, NJ: IEEE. 10.1109/TNNLS.2021.3095068 34280109

[B36] Seeman S. C. Campagnola L. Davoudian P. A. Hoggarth A. Hage T. A. Bosma-Moody A.. (2018). Sparse recurrent excitatory connectivity in the microcircuit of the adult mouse and human cortex. Elife 7:e37349. 10.7554/eLife.37349 30256194 PMC6158007

[B37] Stewart K. Orchard G. Shrestha S. B. Neftci E. (2020). Online few-shot gesture learning on a neuromorphic processor. IEEE J. Emerg. Sel. Topics Circuits Syst. 10, 512–521. 10.1109/JETCAS.2020.3032058

[B38] Südhof T. C. (2018). Towards an understanding of synapse formation. Neuron 100, 276–293. 10.1016/j.neuron.2018.09.040 30359597 PMC6226307

[B39] Sun Q. Liu Y. Chua T. S. Schiele B. (2019). Meta-transfer learning for few-shot learning, in Proceedings of the IEEE/CVF Conference on Computer Vision and Pattern Recognition. (Piscataway, NJ: IEEE), 403–412. 10.1109/CVPR.2019.00049

[B40] Suvarna Y. Maity N. Shivamurthy M. C. (2016). Emerging trends in retrograde signaling. Mol. Neurobiol. 53, 2572–2578. 10.1007/s12035-015-9280-5 26081150

[B41] Tavanaei A. Ghodrati M. Kheradpisheh S. R. Masquelier T. Maida A. (2019). Deep learning in spiking neural networks. Neural Netw. 111, 47–63. 10.1016/j.neunet.2018.12.002 30682710

[B42] Tavanaei A. Maida A. (2019). BP-STDP: Approximating backpropagation using spike timing dependent plasticity. Neurocomputing 330, 39–47. 10.1016/j.neucom.2018.11.014

[B43] Tavanaei A. Maida A. S. (2015). A minimal spiking neural network to rapidly train and classify handwritten digits in binary and 10-digit tasks. Int. J. Adv. Res. Artif. Intell. 4, 1–8. 10.14569/IJARAI.2015.040701

[B44] Thiele J. C. Bichler O. Dupret A. (2018). Event-based, timescale invariant unsupervised online deep learning with STDP. Front. Comput Neurosci. 12, 46. 10.3389/fncom.2018.00046 29962943 PMC6010570

[B45] Ullman S. (2019). Using neuroscience to develop artificial intelligence. Science 363, 692–693. 10.1126/science.aau6595 30765552

[B46] Vogt N. (2018). Machine learning in neuroscience. Nat. Methods. 15, 33–33. 10.1038/nmeth.4549

[B47] Wan L. Zeiler M. Zhang S. Le Cun Y. Fergus R. (2013). Regularization of neural networks using drop-connect, in International Conference on Machine Learning (Atlanta, GA), 1058–1066.

[B48] Wang Y. Yao Q. Kwok J. T. Ni L. M. (2020). Generalizing from a few examples: a survey on few-shot learning. ACM Comput. Surveys. 53, 1–34. 10.1145/3386252

[B49] Waters J. Helmchen F. (2006). Background synaptic activity is sparse in neocortex. J. Neurosci. 26, 8267–8277. 10.1523/JNEUROSCI.2152-06.2006 16899721 PMC6673816

[B50] Wu J. Chua Y. Zhang M. Yang Q. Li G. Li H. (2019). Deep spiking neural network with spike count based learning rule, in International Joint Conference on Neural Networks (IJCNN). (Piscataway, NJ: IEEE), 1–6. 10.1109/IJCNN.2019.885238034288879

[B51] Wu J. Zhao Z. Sun C. Yan R. Chen X. (2020). Few-shot transfer learning for intelligent fault diagnosis of machine. Measurement 166, 108202. 10.1016/j.measurement.2020.10820234763886

[B52] Wu Y. Zhao R. Zhu J. Chen F. Xu M. Li G.. (2022). Brain-inspired global-local learning incorporated with neuromorphic computing. Nat. Commun. 13, 1–14. 10.1038/s41467-021-27653-2 35013198 PMC8748814

[B53] Wyss R. König P. Verschure P. F. (2003). Invariant representations of visual patterns in a temporal population code. Proc. Natl. Acad. Sci. U. S. A. 100, 324–329. 10.1073/pnas.0136977100 12502790 PMC140966

[B54] Yang W. Carrasquillo Y. Hooks B. M. Nerbonne J. M. Burkhalter A. (2013). Distinct balance of excitation and inhibition in an interareal feedforward and feedback circuit of mouse visual cortex. J. Neurosci. 33, 17373–17384. 10.1523/JNEUROSCI.2515-13.2013 24174670 PMC3812505

[B55] Zhang M. Wang J. Zhang J. Belatreche A. Wu J. Chua Y.. (2020). Spike-timing-dependent back propagation in deep spiking neural networks. arXiv[Preprint].arXiv:2003.11837 (2020).

[B56] Zhou Q. Ren C. Qi S. (2020). An imbalanced R-STDP learning rule in spiking neural networks for medical image classification. IEEE Access 8, 224162–224177. 10.1109/ACCESS.2020.3044646

